# Comparison of Different Surgical Techniques for Osia^®^ System Implantation—Experience from Two European Clinical Centers

**DOI:** 10.3390/jcm15010057

**Published:** 2025-12-21

**Authors:** Wojciech Gawęcki, Ann-Kathrin Rauch, Marta Pietraszek, Maria Jaworska, Susan Arndt

**Affiliations:** 1 Department of Otolaryngology and Laryngological Oncology, Poznan University of Medical Sciences, 60-355 Poznań, Poland; pietm@o2.pl (M.P.); maria.jaworska@usk.poznan.pl (M.J.); 2 Department of Otorhinolaryngology, Medical Center–University of Freiburg, Faculty of Medicine, University of Freiburg, 79106 Freiburg, Germany; ann-kathrin.rauch@uniklinik-freiburg.de (A.-K.R.); susan.arndt@uniklinik-freiburg.de (S.A.); 3 Doctoral School, Poznan University of Medical Sciences, 60-812 Poznań, Poland

**Keywords:** bone conduction devices, bone conduction implants, Osia^®^, hearing loss, single-sided deafness, surgical technique

## Abstract

**Background/Objectives**: This study aimed to compare two surgical techniques for Osia^®^ system implantation performed at two European clinical centers: Poznań (Poland) and Freiburg (Germany). **Methods**: The study included 83 patients who underwent Osia^®^ OSI200 and OSI300 implantation (89 implants). The analysis focused on surgical technique, postoperative healing, and long-term skin integrity and aesthetic outcomes. **Results**: The centers differed in their surgical approaches, particularly regarding skin incision design and bone preparation. Most patients experienced no complications. Implant explantation was required in two cases, and one patient with recurrent seroma underwent revision surgery. Both centers achieved excellent postoperative skin integrity, with minimal scar visibility in most patients. Patients treated in Freiburg showed significantly better outcomes in terms of retroauricular bump visibility or palpability (*p* < 0.05) and postoperative pain (*p* < 0.05). Conversely, patients operated on in Poznań reported numbness less frequently (*p* < 0.05). **Conclusions**: Osia^®^ system implantation is a safe and well-tolerated procedure, with postoperative complications occurring in only a small proportion of cases. The Freiburg technique appears to reduce visibility and palpability of retroauricular bump and postoperative pain, but may slightly increase the risk of numbness and, in some cases, lead to a more visible scar compared to the Poznań approach. Optimal outcomes may be achieved by combining elements of both surgical techniques.

## 1. Introduction

Bone conduction implants (BCIs) are a well-established therapeutic option for patients with unilateral or bilateral conductive and mixed hearing loss, as well as those with single-sided deafness (SSD) [1,2,3]. The first successful implantation was reported by Tjellström and Granström in 1977 [4]. Since then, a variety of BCI systems have been introduced, which are typically classified into passive and active types [3,5,6].

In active bone conduction systems, only the external sound processor is positioned outside the body, typically posterior and superior to the auricle. The remaining components, including the transducer, are implanted directly in or on the patient’s bone, allowing for high-quality sound transmission. The processor is magnetically coupled to the implanted component through the intact skin, thereby maintaining skin integrity [1,5,7]. This concept was developed to address the limitations of passive devices, including skin discontinuity and unsatisfactory aesthetic outcomes typical of passive percutaneous systems, as well as soft tissue attenuation and pressure-related skin complications commonly seen in passive transcutaneous systems.

Currently, three active bone conduction implant systems are available on the market: two electromagnetic devices—Bonebridge^®^ (MED-EL, Innsbruck, Austria) [7] and Sentio^®^ (Oticon, Nice, France) [8]—and one piezoelectric system, Osia^®^ (Cochlear Ltd., Sydney, Australia) [1,9,10]. The Osia^®^ system received CE approval in 2019 and was initially released to eight selected clinics across Europe as an early market release. Its safety and efficacy have been demonstrated in multiple studies [11,12,13,14,15,16,17,18]. To streamline the surgical procedure, the device was subsequently modified and introduced as the Osia^®^ OSI200 system, which received FDA approval in the USA in 2019 and CE marking in Europe in 2021. Numerous studies have confirmed the safety of the surgical procedure and the excellent audiological outcomes associated with this device [1,2,3,19,20,21,22]. In June 2024, a new version of the device, Osia^®^ OSI300, was introduced to the market. It differs from its predecessor in the design of the internal magnet, which imposes fewer restrictions during MRI scans than the previous model. However, the external structure of the implant and the surgical technique remain identical to those of the Osia^®^ OSI200. Although the surgical procedure for Osia^®^ OSI200 and OSI300 is thoroughly detailed in the Osia^®^ surgical guides [9,10] several variations have been reported in the literature [23,24,25,26,27,28,29].

This study aims to compare the outcomes of skin integrity and the aesthetic results of two surgical techniques for Osia^®^ system implantation as practiced in two European clinical centers (Poznań, Poland, and Freiburg, Germany).

## 2. Materials and Methods

### 2.1. Study Design

The study was conducted in two tertiary referral clinical centers (Poznań, Poland, and Freiburg, Germany) and included 83 patients who received the Osia^®^ hearing implant (Osia^®^ OSI200 or Osia^®^ OSI300) between April 2021 and February 2025. In total, 89 Osia^®^ procedures were performed in these patients (73 Osia^®^ OSI200 and 16 Osia^®^ OSI300), which were analyzed in terms of: the course of the surgery itself, the course of the healing process after the procedure, the long-term skin integrity and aesthetic outcomes and aspects related to the selection of force and changes of the magnet. The information was obtained from the retrospective analysis of patients’ medical records and telephone interviews conducted from March to May 2025. The study was approved by local Bioethics Committee in Poznań (decision number 539/25) and in Freiburg (decision number 21-1142_1).

### 2.2. Osia^®^ OSI200 and Osia^®^ OSI300 System

The Osia^®^ OSI200 and Osia^®^ OSI300 systems evaluated in this study consist of two implantable components—the BI300 bone conduction implant and either the OSI200 or OSI300 active piezoelectric implant—as well as an external sound processor. The systems are indicated for patients with unilateral or bilateral conductive or mixed hearing loss, as well as for people with single-sided deafness. In cases of mixed hearing loss, the bone conduction pure tone average in the implanted ear must not exceed 55 dB HL [30]. However, the patient’s preoperative bone conduction curve should not be directly at the power limit of the Osia^®^ but should contain a reserve in case of potential further hearing deterioration [31].

### 2.3. Patients

The analyzed group consisted of 83 patients aged 12 to 79 years (average 47 years), including 43 women and 40 men. The most common audiological indication for implantation was bilateral mixed or conductive hearing loss, found in 40 patients. The remaining patients had unilateral mixed or conductive hearing loss (8 patients), unilateral deafness (17 patients), or mixed or conductive hearing loss in the implanted ear, and sensorineural hearing loss of varying degrees in the contralateral ear (18 patients). The largest group of patients consisted of cases with chronic otitis media (either after previous surgical treatment or in situations where middle ear surgery was contraindicated)—49 patients. Other otologic indications included otosclerosis (after previous unsuccessful surgical treatment) in 6 patients, congenital and acquired stenosis of the external auditory canal in 9 patients, and recurrent inflammation of the external auditory canal in 2 patients. In 17 patients the cause of hearing loss/deafness was other or unclear. Five patients had previously been implanted with Baha^®^ Connect system, one with Baha^®^ Attract, one with Osia^®^ OSI100, and the other with Vibrant Soundbridge^®^. The reasons for their replacement with the Osia^®^ system were recurrent skin reactions (Baha^®^ Connect), insufficient hearing (Baha^®^ Attract), MRI-related issues—patient required multiple MRI scans (Osia^®^ OSI100) and device failure (Vibrant Soundbridge^®^). Implantation was performed on the right side in 38 patients, on the left side in 39 patients, and bilaterally in 6 patients (simultaneously in 3 patients and sequentially in 3 patients).

The details of patients operated in both centers are presented in Table 1.

### 2.4. Preparation for the Procedure

Each patient underwent the typical bone conduction implant qualification procedure, including an otologic and audiological assessment and fitting of the BCI processor on a test headband. All met the otologic and audiological criteria for implantation.

Computed tomography (CT) of the temporal bones was performed at the Poznań clinic only in selected cases, while in Freiburg it was routinely performed. This different approach to CT scanning resulted from the different surgical techniques used in the two centers. In Freiburg, where bone well was routinely performed, it was essential for planning the procedure, whereas in Poznań, where bone was merely polished, it was usually not necessary. However, more than half of the patients (exactly 54%) had already undergone computed tomography of the temporal bones during qualification for the Osia^®^ procedure, usually due to previous ear surgery, and in such cases the examination was not repeated. A head MRI was performed at the Poznań clinic in all cases of single-sided deafness and some other patients, and in Freiburg in all cases with any type of hearing loss. In Freiburg, all patients underwent an MRI examination before implantation of the bone conduction device to rule out any intracranial pathology. This step was deemed necessary due to the significant artifact generated by the Osia^®^ device during MRI examinations, which could potentially hinder the detection of intracranial abnormalities or impede accurate assessment.

### 2.5. Surgery and Fitting

#### 2.5.1. Osia^®^ Surgery in Poznań Clinic

**Figure 1:**
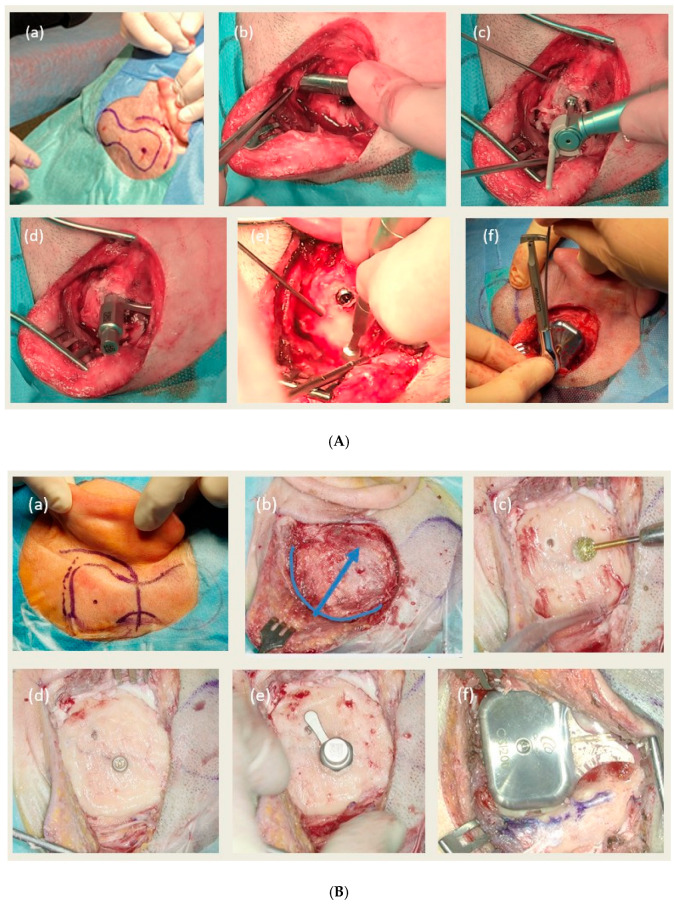
The rules of the surgery in both centers: (**A**) Surgical procedure in Poznań (right side): (**a**) skin incision, (**b**) soft tissue preparation, (**c**) implantation of the BI300 implant, (**d**) inspection of the bone surface, (**e**) bone polishing, (**f**) implantation of the OSI implant. (**B**) Surgical procedure in Freiburg (left side): (**a**) skin incision, (**b**) soft tissue preparation (the blue line shows the incision site of the second layer, and the blue arrow shows the direction of its preparation), (**c**) preparation of the bone well, (**d**) implantation of the BI300 implant, (**e**) inspection of the bone surface, (**f**) implantation of the OSI implant.

All surgeries were performed under general anesthesia by a single experienced otologic surgeon. The surgical technique followed the Cochlear surgical guide [9,10], with a slight modification to the skin incision: a C-shaped incision was made, directed forward and downward from the planned implant site (Figure 1A). The skin incision used in Poznań was a modification of the typical Inferior Postauricular Incision recommended by the manufacturer and described in the Osia surgical guide [9,10]. The modification involved extending this incision in both directions, primarily posteriorly, creating a C-shape and allowing for significantly better exposure of the surgical site. The periosteum was routinely separated from the bone only at the site of the planned BI300 implant and at the pocket for the receiving coil, and at the transducer site it was usually separated only where bone polishing was required. Bone polishing was carried out only when necessary—after the insertion of the BI300 implant and assessment of the bone surface using a dedicated instrument. Only in three cases where large unevenness of the bone surface was observed, preliminary bone polishing was performed before BI300 implantation. The 4 mm long BI300 implant was always used, because no bone reduction was performed before its placement, and the bone at the implantation site was always sufficiently thick. Most patients received three doses of cefuroxime at 8-h intervals, starting with a preoperative dose. Routinely the patients were discharged home the day after the procedure. The first fitting was carried out four weeks after surgery.

#### 2.5.2. Osia^®^ Surgery in Freiburg Clinic

Almost all procedures were performed under general anesthesia by a single experienced otologic surgeon. Only four procedures were performed with the participation of two surgeons. In one case, the procedure was performed under local anesthesia due to multiple comorbidities in the patient (chronic obstructive pulmonary disease, coronary artery disease, diabetes, obesity, kidney disease). The surgical technique followed the Cochlear surgical guidelines; however, some significant modifications were introduced. In Freiburg, a C-shaped retroauricular incision was also used, but unlike the incision used in Poznań, it was extended more cranially, passing over the waist of the OSI 200/300 [23] (Figure 1B). The second significant difference in surgical technique was the routine creation of a bone well for the implant transducer. Prior to Osia^®^ implantation, the thickness of the cortical bone at the intended implant site was assessed preoperatively using computed tomography (CT) or cone beam computed tomography (CBCT) imaging. Based on these measurements, the depth of the bone well was planned to accommodate a 3 mm BI300 implant while allowing maximal recessing of the fixture to achieve an optimal functional and aesthetic outcome and to minimize pressure and stress on the overlying skin. During surgery, the bone well was drilled first according to the preoperative plan, followed by insertion of the BI300 implant. Subsequently, the surrounding bone was smoothed and polished to ensure stable and even implant seating. The bone well was performed as a routine, before insertion of the BI300 implant, except for two cases with limited bone thickness (≤3 mm) and two who had previously received a BI300 implant. Here, only surface polishing was performed. The 3 mm BI300 implant was routinely used, except for two patients who had previously been implanted with a 4 mm BI300 implant. The choice of a 3 mm long implant was related to the surgical technique used, which involved significant bone reduction when preparing the bone well. All patients received intravenous cefuroxime for two days followed by oral cefuroxime for 5 days. Patients were discharged home on the third postoperative day. The first fitting was carried out four weeks after surgery.

The rules of the surgery in both centers are presented in Table 2 and Figure 1.

### 2.6. Evaluated Parameters

The following parameters were evaluated:

1. Course of the surgery itself (surgery time, implant position, soft tissue reduction, bone polishing, any problems or difficulties during surgery)
2. Healing process after the procedure (assessed until the sound processor was connected, which took place four weeks after surgery in both centers)
3. Long-term skin integrity and aesthetic effects—evaluated by the patients themselves and collected by phone interview between March and May 2025 (the follow-up time was 3–48 months, the median follow-up time in Poznań and Freiburg was 17 and 26 months, respectively):
  - incision line/scar visibility—assessed by our own simple scale, scored as follows: 1—completely or almost invisible, 2—visible upon close inspection, 3—clearly visible/striking,
  - presence of a retroauricular bump—assessed by our own simple scale, scored as follows: 1—not palpable, 2—palpable, but no visible prominence, 3—visible, but no interference with glasses (including sunglasses), 4—interference with glasses (including sunglasses),
  - presence of inflammation, pain and numbness of the skin assessed on the IPS (Inflammation, Pain, Skin height/numbness) scale (more information about scoring in Section 3) [32]
4. Aspects related to the selection of force and changes of the magnet.

### 2.7. Statistical Analysis

Statistical analysis was performed using PQStat software, version 1.8. Descriptive statistics and frequency tables were generated, and the normality of data distribution was assessed. The significance level was established at α = 0.05. Comparisons between groups were conducted using the Mann–Whitney test, while categorical variables were compared using the Chi-square test.

## 3. Results

### 3.1. Course of Surgery

In all cases, the surgery was successful, without any significant problems or complications. However, in the Poznań group, in one case with a very fragile dura mater, a small leak of cerebrospinal fluid occurred during inspection of the dura surface. In this case dura was covered with a piece of Tachosil and periosteum which were inserted to the bottom of the hole before insertion of the BI300 implant. Additionally, the antibiotic was changed to ceftriaxone. No further problems were observed in this patient.

The median surgery time in typical surgery (unilateral cases without additional surgical procedure) was 64.5 min in Poznań and 51 min in Freiburg. However, the time differences between individual cases were large (31–110 min) and depended on the anatomical conditions of the patients. The median incision length was 65 mm in Poznań and 70 mm in Freiburg. The median distance of the BI300 implant from the external auditory canal was 55 mm in Poznań and 50 mm in Freiburg. The median implant insertion force in both centers was 40 Ncm. Since the tissue thickness above the receiving coil did not exceed 10 mm in any patient in both centers, there was no need for soft tissue reduction, but in two patients with tissue thicknesses of 9 mm, the implant coil was placed above the periosteum.

At the Poznań clinic, implant position was determined based on the position of the auricle and external auditory canal and then adjusted intraoperatively based on the anatomical conditions (bone curvature, bone irregularities, presence of the emissary vein). This adjustment was performed in 11 (22.4%) procedures (upward or backward displacement). Bone polishing was performed only when necessary and was carried out in 33 (67.3%) procedures. During one Poznań surgery, a Vibrant Soundbridge^®^ implant (MED-EL) with device failure was removed prior to Osia^®^ OSI200 implantation.

At the Freiburg clinic, implant placement was planned based on the position of the auricle and external auditory canal and the preoperative CT or cone beam CT of the temporal bone, and a bone bed of 2–7 mm (mostly 4–5 mm) depth was routinely created before implanting the BI300—36 procedures (90%). The exceptions were two patients whose bone thickness was ≤3 mm and two who had previously received a BI300 implant (one after Baha^®^ Attract and the other during the transition from OSI100 to OSI300). In these patients, the bone was polished instead of drilling a bone bed. In one case, implant position was previously adjusted because of a CSF shunt on the same side of the head.

Detailed data on the course of surgical procedures in both centers are presented in Table 3.

### 3.2. Healing Process After the Procedure

Healing proceeded normally in most patients both in Poznań and Freiburg.

However, in the Poznań group, four cases presented with a small hematoma on the first day after the procedure. The hematomas were successfully removed during dressing changes using pressure and suction. These patients were treated with pressure dressing and received a 7-day course of antibiotics. No further complications occurred, and revision surgery was not required. Additionally, three patients reported significant pain following the procedure, which gradually subsided over the subsequent weeks. Some patients experienced mild pain for a few days postoperatively, along with a slight reduction in skin sensitivity lasting up to several weeks.

In the Freiburg group problems with healing were observed in two patients. Abnormal wound healing was observed in one case (after percutaneous dermatome BCI surgery in medical history), which led to the explantation of Osia^®^ and reimplantation of percutaneous BCI. In another case, a postoperative seroma occurred which required reoperation. In patients with a history of percutaneous BCI implantation (all performed using the dermatome technique), the previous device and abutment were removed in three cases six weeks prior to Osia^®^ surgery and in two cases the Baha^®^ implant was lost before Osia^®^ surgery. In the first patient with a prior percutaneous BCI, the abutment was removed, and six weeks later a rotational flap was used for skin closure with simultaneous Osia^®^ implantation. Postoperatively, a prolonged wound infection developed, eventually necessitating explantation of the Osia^®^. After a healing period of five months, a new percutaneous BCI was implanted. Based on this experience, a two-stage approach was subsequently adopted for the following patient with prior percutaneous BCI: first, removal of the previous implant and closure of the skin using a bilobed flap; second, Osia^®^ implantation six weeks later to allow complete healing of the skin flap. The postoperative course in this patient was uneventful.

### 3.3. Long-Term Skin Integrity and Aesthetic Outcomes (For Bilateral Patients Evaluated for Both Sides Separately)

Long-term results of implanted patients collected in the period from March to May 2025 were obtained from a total of 77 patients (83 surgeries), including 46 patients (47 surgeries) from Poznań and 31 patients (36 surgeries) from Freiburg. Both the Poznań and Freiburg centers demonstrated excellent outcomes in terms of skin integrity, with 100% of patients maintaining intact skin and exhibiting no edema during long-term follow-up. In Poznań, a minor subset of patients (4%) presented with mild erythema. Scar visibility was minimal in most cases, with 98% of patients in Poznań and 86% in Freiburg showing scars that were either completely or nearly invisible, or only detectable upon close examination. Statistically significant differences were found in the frequency of retroauricular bump, pain and numbness. In Poznań, only 4% of patients exhibited no palpable bump, while 66% presented with a palpable but non-visible prominence, and 30% showed a visible bump. In contrast, in Freiburg, 47% of patients had no palpable bump, 50% demonstrated a palpable but non-visible prominence, and only 3% had a visible bump. In one patient operated in Poznań, the Osia^®^ implant was removed two years after implantation—mainly due to auditory artifacts that could not be eliminated despite numerous adjustments and replacement of the sound processor, and because of the unsatisfactory aesthetic effect (visible retroauricular bump). Postoperative pain was reported by 23% of patients in Poznań, although it seldom interfered with implant use. In contrast, no patients in Freiburg reported experiencing pain. Skin numbness occurred more frequently in Freiburg (28%) than in Poznań (9%).

Detailed data on long-term skin integrity and aesthetic outcomes are presented in Table 4.

### 3.4. Aspects Related to the Selection of Force and Changes of the Magnet

In all patients, the magnet strength was determined based on Digital Calibration Link (DCL) measurements and individual subjective feedback.

In the Poznań clinic, the median DCL value measured while the processor was connected was 4.1 mm (range: 2–11 mm). The initially recommended magnet strength had a median of 2 (range: 1–4) and was applied in 27 out of 49 cases (48 patients). However, based on patients’ subjective perceptions, the magnet strength was adjusted to a stronger one or two levels in 21 cases and to a weaker level in 1 case, resulting in a median applied magnet strength of 2 (range: 1–4). Long-term data on magnet strength were available for all 49 cases (48 patients). In 35 of the 49 cases, the magnet strength remained unchanged, but in 14 patients it was further adjusted, being reduced by one level in 9 cases and increased by one level in 5 cases, yielding a final median magnet strength of 2 (range: 1–4).

In the Freiburg clinic, the median DCL value measured while the processor was connected was 5.7 mm (range: 3–10.6 mm). The recommended magnet strength had a median of 3 (range: 1–4) and was applied in 22 out of 39 cases (34 patients; data were not available for one patient). However, based on patients’ subjective perceptions, the magnet strength was adjusted to a stronger level in 14 cases and to a weaker level in 3 cases, resulting in a median applied magnet strength of 3 (range: 1–4). Long-term data on magnet strength were available for 37 out of 40 cases (32 out of 35 patients). In 18 of the 37 cases, the magnet strength remained unchanged, but in 19 cases it was further adjusted, being increased by one or two levels in 7 patients and decreased by one or two levels in 12 patients, resulting in a final median magnet strength of 3 (range: 1–4).

## 4. Discussion

In this study two surgical techniques for Osia^®^ system implantation, practiced in two European clinical centers, were presented. Both the course of surgery and healing, and long-term skin integrity and aesthetic outcomes were compared. Additionally, aspects related to the selection of the magnet strength and its change were analyzed.

### 4.1. Surgery

Osia^®^ system surgeries in both clinics differed significantly in two aspects—the method of soft tissue incision and the method of handling the bone at the implantation site.

#### 4.1.1. Soft Tissue Incision

In Poznań, the surgical team used a C-shaped skin incision carried forward and downward from the planned implant site, which was an own modification of the inferior postauricular incision recommended by the manufacturer. In Freiburg, a two-layer C-shaped incision extending posteriorly over the waist of the implant was used mostly incorporating the previous retroauricular scar. The manufacturer’s surgical guide describes several possible incisions, including a postauricular incision with superior extension, an inferior postauricular incision with extension, and a posterior C-shaped incision [9,10]. All these incisions are located beyond the implant site (according to the manufacturer’s recommendations, the incision should not be over the implant, and what is more, it should be at least 15 mm from its edge). However, the literature describes many types of incisions made over the implant waist or implant transducer [23,25,26,27,28]. The authors of these studies emphasize that such approach optimizes the surgical field while allowing tension-free wound closure. Our study showed that both the incision beyond the implant area (Poznań) and above it (Freiburg) allows for good bone exposure, successful implantation and proper healing in majority of patients.

#### 4.1.2. Handling the Bone at the Implantation Site and Related Aspects

The second significant difference between the procedures in Poznań and Freiburg was the handling of bone at the implant site. In Poznań, the manufacturer’s recommendations were followed, meaning polishing/reduction of bone was performed selectively—only when necessary—after placement of the BI300 implant and evaluation of the bone surface with a dedicated instrument. In the analyzed group, significant bone polishing was necessary in 11 cases (22%), minor in 22 cases (45%), while 16 cases (33%) did not require bone polishing. Previous studies have reported that bone polishing was performed in 41–53% of cases [1,2,25].

In Freiburg, a completely different approach was adopted regarding the bone around the Osia^®^ implant transducer. In all patients where this was possible, a bone bed was routinely created before insertion of the BI300 implant. The goal was to better conceal the implant in the bone, reduce its visibility and palpability after surgery, and decrease the tension of the soft tissues in the surgical area. In the analyzed group, such a procedure was used in 36 cases (90%).

Bone management also influenced the preoperative imaging performed. The technique used in Freiburg required routine preoperative CT to assess bone thickness at the implant site and estimate the depth of the bed, while in Poznań CT was done only in selected cases. Different bone procedures also influenced the choice of the BI300 implant length. As a standard, a 4 mm long implant was used in Poznań, and a 3 mm long implant in Freiburg. Previous studies have also emphasized the importance of bone thickness in determining the appropriate implant length. Willenborg et al. highlighted the role of preoperative CT in assessing bone thickness and anatomical variations, allowing the selection of either 3- or 4-mm implants [17]. Soloperto et al. demonstrated that the decision to use a 3- or 4-mm implant depends on intraoperative assessment of bone thickness [33]. In contrast, Ślęzak et al. reported that all 14 adult patients in their cohort received 4 mm Cochlear BI300 implants without preoperative imaging [34]. Posta et al. studied a pediatric population and suggested that 3 mm BI300 implants are generally suitable for younger pediatric patients, whereas 4 mm implants may be used in older children aged 11–12 years [35].

An interesting observation from our study is that routine bone well creation does not prolong the procedure time. The median surgery duration in Poznań was 64.5 min and was within the range of previously published durations of 40 to 87.2 min [1,2,19,25]. In Freiburg it was only 51 min. The shorter operating time in Freiburg compared to Poznań can be explained by the lack of additional time required for drill preparation during surgery. The drill in Freiburg was always prepared before the procedure, while in Poznań it was prepared during the operation at the surgeon’s request.

#### 4.1.3. Surgical Problems and Intraoperative Complications

In majority of implanted cases in our cohort no intraoperative problems occurred. However, in one patient in the Poznań group a small leak of cerebrospinal fluid occurred during surgery, which was successfully stopped by a piece of Tachosil and periosteum and by insertion of the BI300 implant. In literature other surgical problems were described. Goldstein et al. described an exposure of the dura with bleeding from the transverse sinus. Despite this, the BI300 implant fixture was successfully placed [1]. Also, Ślęzak et al. experienced mild intraoperative bleeding in one patient that was successfully controlled [34].

### 4.2. Healing Process After the Procedure

In our study, healing progressed normally in most patients in both Poznań and Freiburg. However, in the Poznań group, seven patients experienced mild postoperative complications—four cases of small hematomas detected one day after surgery and three cases of significant but temporary pain. All these issues were resolved without the need for reoperation. In contrast, in Freiburg, two cases required reoperation: one due to abnormal wound healing after previous excessive reduction of subcutaneous soft tissue for BCI implantation necessitating Osia^®^ explantation, and another due to a postoperative seroma that required surgical management.

Similarly, in the literature regarding Osia^®^, healing proceeded normally in most cases and only isolated healing problems were described. Goldstein et al. evaluated 44 Osia^®^ 2 implantations and reported two cases of postoperative wound infection, both successfully managed with antibiotics, as well as two postoperative hematomas—one resolving after the application of cold compresses and the other treated by needle aspiration [1]. Similarly, Briggs et al. reported three moderate postoperative events among 29 adult implant recipients: pain in one patient and wound infections in two patients, both effectively treated with antibiotics [2]. Also, Ślęzak et al., using the SCOTNI (semicircular over-the-neck incision) technique in 14 patients, described one patient, who developed delayed wound healing accompanied by a cervical hematoma and skin flap necrosis, requiring revision with a local flap [34]. Four weeks after revision, primary healing was achieved, and the sound processor was fitted successfully [34]. Similarly, Malagutti et al. described a rare case of Osia^®^ extrusion associated with retroauricular wound dehiscence, initially unresponsive to conservative treatment, requiring revision surgery with a local flap and the use of nylon sutures due to a suspected reaction to Vicryl. The wound fully healed after six months, allowing daily device use [36]. In turn, Young et al. reported one patient with an OSI200 extrusion necessitating surgical removal [3].

Overall, these observations indicate that while Osia^®^ implantation is largely safe and well-tolerated, occasional wound- or device-related complications can occur. These findings underscore the importance of careful surgical planning, meticulous intraoperative technique, and thorough patient counseling regarding potential risks.

### 4.3. Long-Term Skin Integrity and Aesthetic Outcomes

Both the Poznań and Freiburg centers achieved excellent postoperative skin integrity, with minimal scar visibility observed in most patients. This indicates that satisfactory cosmetic outcomes were obtained at both sites, although Poznań demonstrated a slightly higher proportion of patients with no or minimal scar visibility. Patients treated in Freiburg demonstrated significantly better results regarding the visibility and palpability of the retroauricular bump as well as postoperative pain, suggesting superior outcomes in these aspects when employing the Freiburg technique with routine bone bed preparation. Conversely, patients operated on in Poznań reported numbness less frequently. We believe that this difference primarily reflects the distinct incision designs used at the two centers.

The literature does not yet provide such a detailed analysis of all aspects related to the skin condition and aesthetic effect after Osia^®^ implantation, and the available descriptions concern problems in individual cases. Cushing et al. reported that among 42 pediatric patients receiving Osia^®^ 2 devices, two developed irritations at the magnet and incision site due to frequent device usage. In both cases, soft tissue irritation and mild skin breakdown were resolved by adding a soft magnet pad to the external processor [37]. Cowan et al., in a prospective multicenter study involving 20 adult recipients of the Osia^®^ 2 System, reported nine adverse events in six subjects during the period from 6 to 24 months post-implantation. Three events—pain behind the implant, skin irritation, and prominence of the posterior inferior edge—were related to both the device and the procedure, while six events were device-related only, including discomfort from sound processor heating, increased tinnitus, frustration, or non-use. Most adverse events were mild, with only two cases of non-use classified as moderate [21]. Brill et al., using an anterior-based retroauricular flap with a J-shaped incision in 21 patients, reported no skin-related complications, infections, or implant extrusion over a follow-up exceeding one year, with only a single patient experiencing pain above the implant coil, which resolved after reducing magnet strength [38].

These observations indicate that minor soft tissue reactions and skin irritation after Osia^®^ implantation may still occur, particularly in patients with high device usage or sensitive skin. Such issues are generally manageable, but underscore the importance of careful postoperative monitoring, patient education, and potential adaptation of external components to reduce tissue stress.

### 4.4. Aspects Related to the Selection of Force and Changes of the Magnet

In the Poznań center, the median DCL value with the processor connected was 4.1 mm, and the median recommended and applied magnet strength was 2. In the Freiburg center, the median DCL value was higher, at 5.7 mm, with a median recommended and applied magnet strength of 3. Comparison of the results indicates that initially, stronger magnets were applied in Freiburg than in Poznań, which may be related to higher DCL values and thicker soft tissues measured during surgery over coil observed in that center. Notably, in both centers, adjustments to magnet strength were required over time in many patients, underscoring the importance of individualized implant fitting based on both DCL measurements and patients’ subjective perceptions. These findings indicate that DCL measurement may provide useful guidance in magnet strength selection, although patient preferences should also be considered.

The literature indicates that the strength of a magnet in any kind of hearing implant is primarily determined by the thickness of the patient’s skin flap. Thicker skin flaps require stronger magnets to ensure a secure connection between the external processor and the internal implant, while thinner flaps necessitate weaker magnets to prevent excessive pressure. Other factors, such as patient age, may also correlate with skin thickness and influence magnet selection. The choice of magnet strength is a collaborative decision between the audiologist, surgeon and patient, aiming to achieve optimal retention while minimizing the risk of skin complications. Magnets that are excessively strong can cause skin breakdown, infection, or implant extrusion, potentially necessitating revision surgery. Preoperative measurement of skin flap thickness is essential to guide magnet selection, and regular postoperative monitoring is necessary to detect redness, swelling, or other skin issues. Any complications should be promptly reported to the audiologist to prevent further adverse outcomes [39,40,41,42].

In conclusion, the findings underscore the critical importance of integrating objective measures, such as DCL, with patients’ subjective assessments in the process of magnet selection. Such a comprehensive, patient-centered approach contributes not only to the functional stability of the implant but also enhances long-term comfort and minimizes the risk of adverse outcomes.

### 4.5. Strengths and Limitations of the Study

The strengths of our study are: (1) all surgeries performed by one of two experienced otosurgeons (WG in Poznań and SA in Freiburg), (2) a homogeneous group of patients—only adults and teenagers (we had no children in the group analyzed), and (3) long-term follow-up. The limitations of the present study are (1) short follow-up time for some patients (less than one year in 12 cases in Poznań and 12 cases in Freiburg, less than 6 months in 2 cases in Poznań and 5 cases in Freiburg), (2) missing long-term magnet strength data for 3 patients in the Freiburg group, (3) differences in the length of hospitalization and antibiotic therapy between centers, (4) subjective nature of the evaluation of long-term outcomes by patients and (5) the lack of comparison with other bone conduction implants.

## 5. Conclusions

Implantation of the Osia^®^ OSI 200 and OSI 300 system is a safe procedure that does not cause any significant problems or complications. Postoperative healing problems occur in a small percentage of cases. The surgical technique used in Freiburg reduces the visibility and palpability of the retroauricular bump caused by the implant transducer under the skin and reduces post-operative pain, but in comparison to the technique used in Poznań it is more likely to cause numbness and in a minority of cases a more visible incision line/scar. Based on our results, we hypothesize that the best results may be achieved by combining both techniques, but this should be verified in a prospective study. Selecting magnet strength based on objective DCL measurements and subjective patient perceptions does not guarantee optimal selection in the long term and requires modification in many patients.

## Figures and Tables

**Table 1:** The details of patients operated in Poznań and Freiburg.

		Poznań	Freiburg	All
Number of patients (ears)		48 (49)	35 (40)	83 (89)
Age (years)	Mean	50	43	47
	Min.	12	14	12
	Max.	76	79	79
Sex	Men	20	20	40
	Women	28	15	43
Hearing loss	Bilateral mixed and conductive	23	17	40
	Unilateral mixed and conductive	4	4	8
	Single-sided deafness	12	5	17
	Other *	9	9	18
Etiology of hearing loss	Chronic otitis media	29	20	49
	Otosclerosis	6	0	6
	Atresia or stenosis of EAC	3	6	9
	Recurrent inflammations of EAC	0	2	2
	Other/unknown	10	7	17
Previous BCI/MEI	Baha^®^ Connect	0	5	5
	Baha^®^ Attract	0	1	1
	Osia^®^ OSI100	0	1	1
	Vibrant Soundbridge^®^	1	0	1
Side of implantation	Right	25	13	38
	Left	22	17	39
	Bilateral simultaneous	0	3	3
	Bilateral sequential	1	2	3

* Other hearing loss—in implanted ear conductive or mixed hearing loss, in the contralateral ear sensorineural hearing loss of varying degrees.

**Table 2:** Typical surgical procedure for Osia^®^ implantation at the clinic in Poznań and at the clinic in Freiburg.

	Poznań	Freiburg
CT/CBCT before surgery	In 31 patients	Always (35 patients)
MRI before surgery	In 17 patients, in all SSD cases	Always (35 patients)
Anesthesia	General	*n* = 39 ears: general *n* = 1 ear: local (sedation)
Soft tissue measurements over the coil	Yes	Yes
Soft tissue incision (see Figure 1)	C-shaped incision, directed forward and downward from the planned implant site	2-layer C-shaped incision incorporating previous postauricular incisions extending posteriorly over the waist of the implant
BI300 implant—length	4 mm	*n* = 38 ears: 3 mm *n* = 2 ears: pre-existing 4 mm BI300 retained (after Baha^®^ Attract and Osia^®^ OSI100)
Bone polishing	only when necessary—after the insertion of the BI300 implant and assessment of the bone surface using a dedicated instrument *	*n* = 36 ears: bone well *n* = 4 ears: only surface polishing
Muscle suture	Optime 2/0	Vicryl 2/0
Subcutaneous suture	Optime 3/0	Vicryl 3/0
Skin suture	Filapeau 3/0	Vicryl rapid 3/0
Perioperative antibiotic	Intravenous cefuroxime 3 doses **	Intravenous cefuroxime for two days followed by oral cefuroxime for 5 days

* In 3 cases with large unevenness of the bone surface initial bone polishing was performed before BI300 implantation. ** In some cases prolongated antibiotic therapy was used (details in Section 3).

**Table 3:** Differences in the procedure between both centers.

		Poznań	Freiburg	
Number of implantations	*n*	49	40	
Comparisons between groups		median (range)	median (range)	Mann-Whitney Test *p*-value
Surgery time *	[min]	64.5 (45–110)	51 (31–96)	0.0002
Soft tissue thickness—over coil	[mm]	5 (3–9)	6 (4–9)	0.0001
Incision length	[mm]	65 (55–80)	70 (50–80)	0.7935
Distance BI300—the center of the ear canal	[mm]	55 (50–65)	50 (40–70)	0.0019
BI300 torque	[Ncm]	40 (30–50)	40 (30–50)	0.8130
Categorical variables		*n* (%)	*n* (%)	Chi-square test *p*-value
Position of BI300	As planned	38 (78%)	37 (93%)	0.0144
	Changed upwards	9 (18%)	0 (0%)	
	Changed posteriorly	2 (4%)	1 (2%)	
	Previous BI300 implant used	0 (0%)	2 (5%)	
Soft tissue reduction	No	49 (100%)	40 (100%)	1.0000
	Yes	0 (0%)	0 (0%)	
Coil position	Under periosteum	48 (98%)	39 (98%)	0.8844
	Above periosteum	1 (2%)	1 (2%)	
Bone reduction	No reduction	16 (33%)	0 (0%)	<0.0001
	Limited bone polishing (after BI300 implantation)	22 (45%)	4 (10%)	
	Significant bone polishing (after BI300 implantation)	11 (22%)	0 (0%)	
	Bone well as routine (before BI300 implantation)	0 (0%)	36 (90%)	

* This applies to cases of unilateral implantation without additional simultaneous otologic or laryngological procedures.

**Table 4:** Long-term skin integrity and aesthetic outcomes in Poznań and Freiburg.

		Poznań	Freiburg	Chi-Square Test *p*-Value
Median follow-up time (months)		17 (Q1 = 12; Q3 = 31)	26 (Q1 = 10; Q3 = 37)	
Number of surgeries with available long-term results data		47	36	
Incision line/scar visibility	1—Completely or almost invisible	30 (64%)	22 (61%)	0.1065
	2—Visible upon close inspection	16 (34%)	9 (25%)	
	3—Clearly visible/striking	1 (2%)	5 (14%)	
Retroauricular bump	1—Not palpable	2 (4%)	17 (47%)	<0.0001
	2—Palpable, but no visible prominence	31 (66%)	18 (50%)	
	3—Visible, but no interference with glasses (including sunglasses)	14 (30%)	1 (3%)	
	4—Interference with glasses (including sunglasses)	0 (0%)	0 (0%)	
IPS—inflammation – skin integrity	intact—0	47 (100%)	36 (100%)	1.0000
	not intact—2	0 (0%)	0 (0%)	
IPS—inflammation – erythema	none—0	45 (96%)	36 (100%)	0.2102
	present—1	2 (4%)	0 (0%)	
IPS—inflammation – oedema	none—0	47 (100%)	36 (100%)	1.0000
	present—1	0 (0%)	0 (0%)	
IPS—pain	none—0	36 (77%)	36 (100%)	0.0077
	yes, but not resulting in diminished usage—1	9 (19%)	0 (0%)	
	yes, and resulting in diminished usage—2	2 (4%)	0 (0%)	
IPS—skin numbness	not present—0	43 (91%)	24 (67%)	0.0490
	present, but not resulting in diminished usage—1	4 (9%)	10 (28%)	
	present, and resulting in diminished usage—2	0 (0%)	0 (0%)	
	not assessed	0 (0%)	2 (5%)	

## Data Availability

The data presented in this study are available on request from the corresponding author.

## Abbreviations

The following abbreviations are used in this manuscript: BCI—bone conduction implant, CBCT—cone beam computed tomography, CE—Conformité Européenne (European Conformity), CT—computed tomography, DCL—Digital Calibration Link, EAC—external auditory canal, FDA—Food and Drug Administration, IPS—Inflammation, Pain, Skin height/numbness, MEI—middle ear implant, MRI—magnetic resonance imaging, SCOTNI—semicircular over-the-neck incision, SSD—single-sided deafness.
